# Identification of parameters and formulation of a statistical and machine learning model to identify *Babesia canis* infections in dogs using available ADVIA hematology analyzer data

**DOI:** 10.1186/s13071-022-05163-4

**Published:** 2022-01-29

**Authors:** Tera Pijnacker, Richard Bartels, Martin van Leeuwen, Erik Teske

**Affiliations:** 1 Department of Clinical Sciences, Utrecht University, Utrecht, The Netherlands; 2 Digital Health, University Medical Center Utrecht, Utrecht, The Netherlands

**Keywords:** ADVIA, *Babesia canis*, Blood smear, Machine learning

## Abstract

**Background:**

Canine babesiosis is an important tick-borne disease in endemic regions. One of the relevant subspecies in Europe is *Babesia canis*, and it can cause severe clinical signs such as hemolytic anemia. Apart from acute clinical symptoms dogs can also have a more chronic disease development or be asymptomatic carriers. Our objective was to identify readily available ADVIA hematology analyzer parameters suggestive of *B. canis* parasitemia in dogs and to formulate a predictive model.

**Methods:**

A historical dataset of complete blood count data from an ADVIA hematology system with blood smear or PCR confirmed parasitemia cases was used to obtain a model by conventional statistics (CS) methods and machine learning (ML) using logistical regression and tree methods.

**Results:**

Both methods identified that important parameters were platelet count, mean platelet volume and percentage large unstained cells. We were able to formulate a CS model and ML model to screen for *Babesia* parasitemia in dogs with a sensitivity of 84.6% (CS) and 100% (ML), a specificity of 97.7% (CS) and 95.7% (ML) and a positive likelihood ratio (LR+) of 36.78 (CS) and 23.2 (ML).

**Conclusions:**

This study introduces two methods of screening for *B. canis* parasitemia on readily available data from ADVIA hematology systems. The algorithms can easily be introduced in laboratories that use these analyzers. When the algorithm marks a sample as ‘suggestive’ for *Babesia* parasitemia, the sample is approximately 37 times more likely to show *Babesia* merozoites on blood smear analysis.

**Graphical Abstract:**

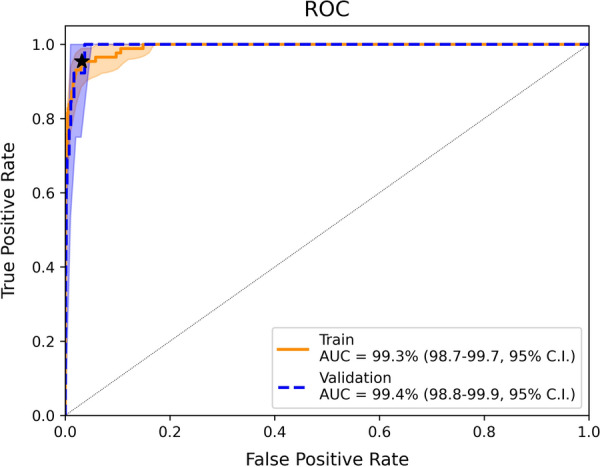

## Introduction

Canine babesiosis is a tick-borne disease caused by species of the protozoan genus *Babesia* [1]. One of these species, *Babesia canis*, is described across most of Europe [2–6]. Its vector is *Dermacentor reticulatus* [7]. Clinical signs of canine babesiosis are variable but can consist of pale mucous membranes, weakness, petechiae and epistaxis [8]. Common hematological abnormalities are mild to moderate anemia, thrombocytopenia, leukopenia with neutropenia and/or lymphopenia [8]. Biochemical abnormalities are also common and can consist of hypoalbuminemia, elevation of liver enzymes, hyperlactatemia, hyperphosphatemia, hypertriglyceridemia, hypoglycemia and (both prerenal and postrenal) azotemia [8].

Diagnosis of an active (parasitemic) *B. canis* infection can be done by light microscopy evaluation of a blood smear. Detection of *B. canis* in stained blood smears has been the standard diagnostic technique for many years. This method is reliable when a moderate to high parasitemia is present [5, 9]. Currently, PCR detection of *Babesia* spp. has become the mainstay of diagnosis because of high sensitivity and more reliable identification of the causative *Babesia* species infecting the dog [9]. However, PCR testing usually takes a few days before results are available, and the acute infections that are often seen in clinical *B. canis* patients make a timely diagnosis of vital importance. The prognosis of *B. canis* ranges from poor to good, depending on the severity of the infection and the time between infection, diagnosis and treatment [1].

Hematology is important in the diagnosis of *B. canis*, as anemia and thrombocytopenia combined with a compatible history should alert clinicians to active babesiosis. As the clinical signs of babesiosis are not always very specific, a warning system based on routine hematology bloodwork would offer advantages. Diagnosing babesiosis by recognizing patterns becomes even more important in non-endemic countries where the prevalence is very low and only imported cases are present. ADVIA hematology analyzers are widely used in larger (veterinary) laboratories [10]. ADVIA hematologic patterns are used in human medicine to guide toward hematological diagnosis, for example, thalassemia, acute myeloid leukemia and megaloblastic anemia [11].

Machine learning (ML) belongs to the area of artificial intelligence. It has been utilized extensively in the medical field as a tool to aid in the diagnosis of medical conditions and make diagnostic predictions [12]. Recently, it also made its entrance into veterinary research [13–16].

The aim of this study was to identify readily available ADVIA hematological parameters suggestive of *B. canis* parasitemia in dogs in a non-endemic region and to compare a model obtained by conventional statistics with a model obtained by machine learning.

## Materials and methods

### Datasets

Two datasets, one for model building and one for validation, were constructed from a search in the Utrecht University Veterinary Diagnostic Laboratory patient files. The modeling dataset contained all dogs that were found to have a *Babesia* parasitemia from 2002 to 2013. A total of 87 dogs with *Babesia* parasitemia, confirmed by blood smear analysis, were enrolled. Data of control dogs (*n* = 1144) were collected from November 2010 through January 2011. In only 63 dogs with *Babesia* and 294 control dogs, all parameters were measured. In the other dogs no reticulocyte parameters were measured.

The validation dataset contained 13 dogs that tested positive for *B. canis* from 2017 up until June 2020. Data from control dogs (*n* = 5649, with 5540 unique patients) were collected from January 2017 through September 2018. Also, dogs in which *Anaplasma phagocytophilum* was found (*n* = 29), from 2017 through June 2020, were present in this control group.

In all dogs of both datasets, 214 different ADVIA parameters related to erythrocytes, reticulocytes, platelets and leukocytes were recorded. In the 2002–2013 period, blood was analyzed with the ADVIA 120 and in the 2017–2020 period with the ADVIA 2120i.

### Building a model with conventional statistics

As a first step in the modeling dataset the means and data within 1 and 2 standard deviations of the mean (1 SD, 2 SD) were calculated for each feature for the *Babesia* group and the control group. Next, those parameters were identified in which the percentage of *Babesia* dogs was most outside the range of mean ± 1 SD or mean ± 2 SD of the control dogs. Following this, the area under the curve (AUC), optimal cutoff value, sensitivity, specificity and positive likelihood ratio (LR+) were calculated for each of these parameters using receiver-operating characteristic (ROC) curves. Then, those parameters that had an AUC > 0.70 were combined into one model to increase diagnostic accuracy, and the sensitivity, specificity and LR+ were calculated for each of these combinations. Finally, the model was used on the validation dataset.

A commercially available software package (SPSS 27.0, IBM SPSS Statistics for Windows, Armonk, NY, USA) was used for data analysis. ROC curves as well as calculation of the AUC were made with commercially available MedCalc^®^ Statistical Software version 20.009 (MedCalc Software Ltd, Ostend, Belgium).

### Machine learning

A classification model was trained on a supervised learning task, $f\left(\overrightarrow{x}\right)= y.$ Here, $f$ represents the model, $y$ is the (binary) label indicating whether the subject has a *B. canis* infection, and $\overrightarrow{x}$ are the input features (i.e. a selection of ADVIA parameters). Several different classifiers—logistic regression, decision tree, random forest and XGBoost [17]—were trained. The tree-based models (decision tree, random forest and XGBoost) can capture non-linear relations in the data.

All selected ADVIA parameters, without any further preprocessing, are used in the tree-based classifiers. For the logistic regression data were first scaled by subtracting the mean and dividing by the standard deviation, i.e. using a standard scaler, after which the *K* best predictors—identified by univariate feature selection—were used as input features. Here, *K* was treated as a hyperparameter. A hyperparameter is a configuration parameter of the model that is not directly learned from data as opposed to model parameters such as the coefficients of a logistic regression.

**Fig. 1:**
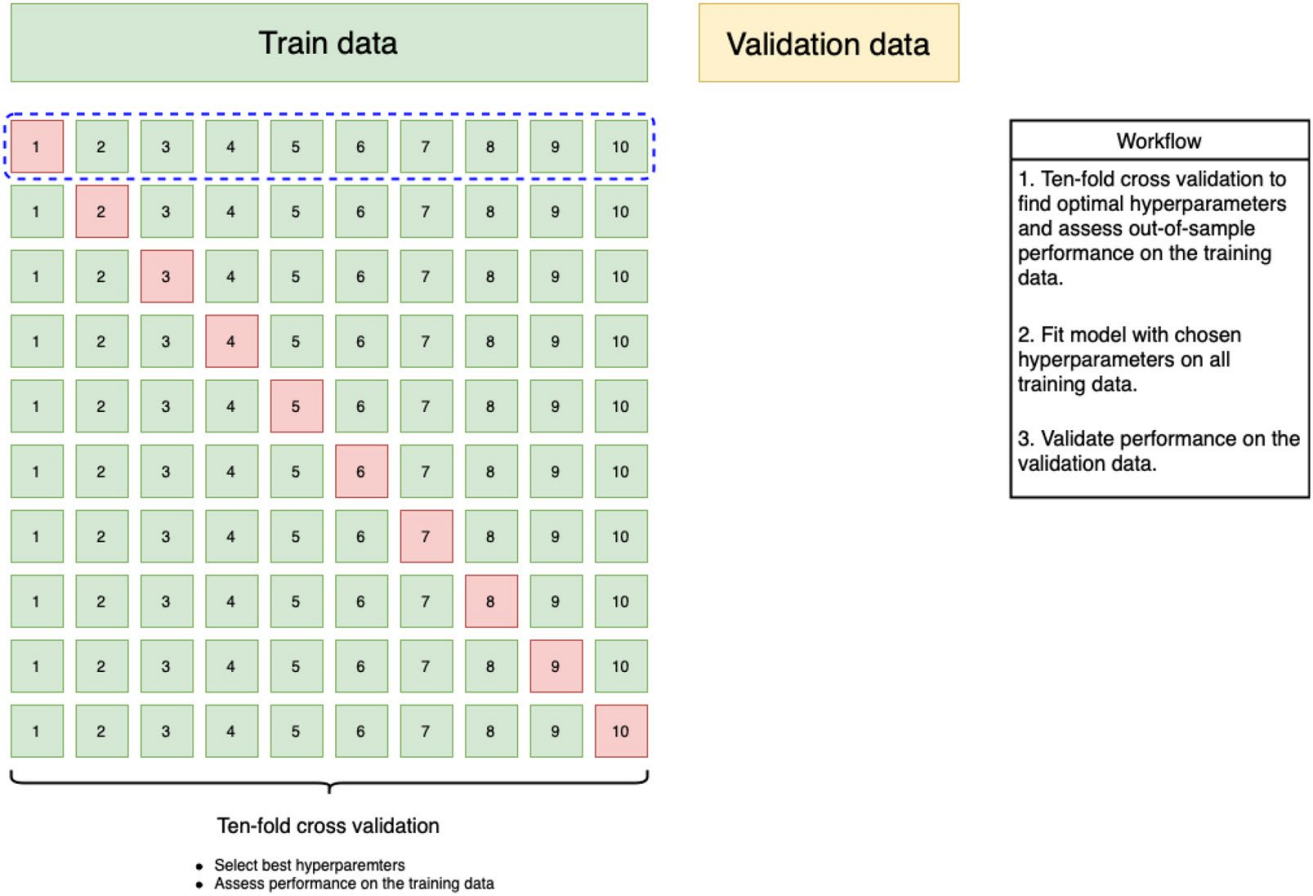
Schematic representation of the machine-learning workflow. Ten-fold cross-validation is used to assess out-of-sample performance and tune hyperparameters for each classifier. In each iteration (e.g. blue dashed box) nine folds are used for training (green) and one fold is used to assess out-of-sample performance. Next, the classifier with its optimal hyperparameters is fit on all training data before finally evaluating its performance on the validation dataset

The train (validation) set contains 1144 (5649) negative samples and 87 (13) positive samples (see section on the dataset). First, ten-fold cross validation on the training data was applied to tune hyperparameters and estimate out-of-sample performance. In the cross-validation procedure the training data are split into ten folds. A model was trained on nine folds, and its performance was assessed on the unseen tenth fold. This procedure was repeated ten times to derive performance metrics based on the training data. Hyperparameter tuning, i.e. optimizing the configuration parameters of each classifier such as the maximum depth of the tree-based classifiers, was done using HyperOpt [18]. HyperOpt uses Bayesian optimization to efficiently try new hyperparameters based on their expected performance, which was measured through the AUC. All experiments were logged in MLflow, and optimal hyperparameters were chosen for each classifier based on the AUC obtained from the cross-validation procedure. Results presented for the training data are for the optimal set of hyperparameters of each classifier. Next, each classifier with its chosen hyperparameters was trained on the full training data and then evaluated on the validation data to assess the generalization error. An overview of the workflow is provided in Fig. 1.

As a threshold for positive predictions, we used the value corresponding to a 95% sensitivity as extracted from the ROC curve; 95% confidence intervals (CI) on the accuracy, sensitivity and specificity are computed by bootstrapping the data 1000 times. Bootstrapping results are not presented for the sensitivity of the validation data, since they only contain a limited number of positive samples.

All data preprocessing, model training and evaluation are performed in Python 3.7 using the packages MLflow 1.11.0, NumPy 1.18.2, pandas 1.0.3, scikit-learn 0.22.2, shap 0.39.0 [19] and XGBoost 1.2.0.

Methods and results are reported in accordance with “MINimum Information for Medical AI Reporting” (MINIMAR) [20], a recently proposed standard for medical artificial intelligence (AI) reporting. Analysis code for the machine learning models is made publicly available via GitHub.

## Results

### Conventional statistics

After calculating means and 1 SD and 2 SD for each of the 214 different parameters related to erythrocytes, reticulocytes, platelets and leukocytes, in the modeling data set, those parameters were identified of which > 30% of the values of the *Babesia* dogs were outside 1 SD of the mean of the control dogs (Table 1). For these parameters ROC curves were drawn, and parameters with a high AUC (> 0.70) were selected and sensitivity, specificity and LR + were calculated (Table 2). To increase the diagnostic accuracy several combinations of parameters were selected (Table 3). Three combinations had a high LR+ : (i) platelet count (PLT) < 102 and a percentage of large unstained cells (%LUC) > 1.8 (LR+  = 39.00), (ii) PLT < 102, platelet dry mass distribution width (PMDW) > 1.09 and %LUC > 1.8 (LR+  = 46.58) and (iii) PLT < 102, mean platelet volume (MPV) > 14 and %LUC > 1.8 (LR+  = 62.43).

**Table 1:** Parameters for which > 30% of the values of *Babesia* dogs were outside 1 SD of mean of control dogs were identified

Test	%		< or >	*n*
	1 SD	2 SD		
|RBC (× 10E12 cells/l)|	43.7	5.8	<	87
HGB (mmol/l)	34.5	3.5	<	87
|HCT (l/l)|	42.5	5.8	<	87
|%LUC (%)|	60.9	33.3	>	87
MN_y_peak ([no units])	80.5	50.6	<	87
lob_Index ([no units])	79.3	3.5	>	87
pcnt_low_retics (%)	39.7	0.0	>	63
pcnt_med_retics (%)	33.3	0.0	<	63
retics_cells_thresh ([no units])	44.4	0.0	>	63
med_retic_thresh ([no units])	82.5	0.0	>	63
high_retic_thresh ([no units]	100.0	0.0	>	63
retic_MCV (fl)	34.9	0.0	<	63
retic_HDW (mmol/l)	36.5	12.7	>	63
retic_H_mean (fmol	31.8	1.6	<	63
% abnormal_cells ([no units])	42.9	18.4	>	87
pcnt_high_px (%)	36.8	4.6	<	87
Lymph_noise_valley	32.2	11.5	>	87
|IRF-M + H (%)|	39.7	0.0	<	63
|MCV_rm_delta (fl)|	41.3	3.2	<	63
HDW_rm_delta (mmol/l)	36.5	7.9	>	63
|CH_rm_delta (fmol)|	41.3	12.7	<	63
CHDW_rm_delta (fmol)	48.4	12.9	>	63
|%macro_r ([no units])|	49.2	0.0	<	63
%lowCH_m ([no units])	28.6	7.9	>	63
|%highCH_r ([no units])|	41.3	0.0	<	63
|RBC_2-D_count (× 10E12 cells/l)|	43.7	5.8	<	87
PLT (× 10E09 cells/l)	98.9	0.0	<	87
MPV (fl)	89.7	59.8	>	87
|MPC (g/l)|	74.7	40.2	<	87
PCDW (g/l)	41.4	1.2	>	87
MPM (pg)	58.6	18.4	>	87
|PMDW (pg)|	89.7	63.2	>	87
RBC_Ghosts (× 10E12 cells/l)	30.3	18.2	<	65
BaroxNRBCCount ([no units])	31.0	0.0	>	87
endCurveMu ([no units])	28.7	8.1	>	87

*N* indicates the number of dogs with *Babesia* in which parameters was measured. For explanation abbreviations see Table 1 Addenda

**Table 2:** Selected individual parameters and calculated cutoff values based on ROC curves

	*N* =	AUC	Sensitivity (%)	Specificity (%)	LR+
PLT (≤ 101 × 10E09 cells/l)	1231	0.966	98.85	90.47	10.37
MPV (> 14 fl)	1231	0.938	95.40	84.70	6.24
Lob_Index (> 2.69)	1231	0.869	81.61	97.50	32.64
MN-y-peak (≤ 10.5)	770	0.917	80.46	95.54	18.04
High_retic_thresh (> 70)	1231	0.708	100	58.42	2.41
PMDW (> 1.09 pg)	1231	0.939	97.70	75.87	4.05
%Luc (> 1.8)	1231	0.929	89.66	88.72	7.95
MPC (≤ 200 g/l)	1231	0.890	82.76	81.56	4.49

**Table 3:** Combinations of parameters to increase diagnostic accuracy in modeling dataset

	Sensitivity	Specificity	LR+
PLT < 102 and PMDW > 1.09	96.6	93.0	13.80
PLT < 102 and MPV > 14	94.3	94.3	16.54
PLT < 102 and %Luc > 1.8	89.7	97.7	39.00
PLT < 102 and PMDW > 1.09 and MPV > 14	93.1	94.8	17.90
PLT < 102 and PMDW > 1.09 and %Luc > 1.8	88.5	98.1	46.58
PLT < 102 and MPV > 14 and %Luc > 1.8	87.4	98.6	62.43

The parameters identified in the modeling data set as having a high AUC (Table 2) were used in the validation set. The known prevalence for *B. canis* in this set was 0.23%. Using this prevalence, the sensitivity and specificity and positive predictive values (PV+) were calculated for each of these parameters (Table 4). The single parameter with highest PV+ was %LUC > 1.8 (PV+  = 3.1%). This was repeated for the combination of parameters found to have the highest diagnostic accuracy in the modeling data set. The combination of PLT < 102 and %LUC > 1.8 had one of the highest PV + (7.7%) (see Table 5). Combining with an extra parameter did not lead to a significant increase of PV+, while on the other hand the sensitivity declined.

**Table 4:** Selected individual parameters evaluated in validation dataset with prevalence of *B. canis* of 0.23%

*N* = 5663	Sensitivity (%)	Specificity (%)	LR+	PV+ (%)
PLT (≤ 101 × 10E09 cells/l)	100	89.4	9.43	2.1
MPV (> 14 fl)	84.6	78.4	3.92	0.9
Lob_Index (> 2.69)	76.9	33.6	1.16	0.3
MN-y-peak (≤ 10.5)	100	1.5	1.02	0.2
High_retic_thresh (> 70)	61.5	58.1	1.47	0.3
PMDW (> 1.09 pg)	92.3	77.2	4.05	0.9
%Luc (> 1.8)	84.6	93.9	13.87	3.1
MPC (≤ 200 g/l)	61.5	69.0	1.98	0.5

**Table 5:** Selected combinations of parameters evaluated in validation dataset with prevalence of *B. canis* of 0.23%

*N* = 5663	Sensitivity (%)	Specificity (%)	LR +	PV+ (%)
PLT < 102 and PMDW > 1.09	92.3	91.3	10.61	2.4
PLT < 102 and MPV > 14	84.6	92.0	10.58	2.4
PLT < 102 and %Luc > 1.8	84.6	97.7	36.78	7.7
PLT < 102 and Lob_Index > 2.69	76.9	93.6	12.02	2.7
PLT < 102 and MPC (≤ 200 g/l)	61.5	93.8	9.92	2.2
PLT < 102 and MN_y_Peak (≤ 10.5)	100	89.6	9.62	2.8
PLT < 102 and PMDW > 1.09 and MPV > 14	84.6	92.5	11.28	2.5
PLT < 102 and PMDW > 1.09 and %Luc > 1.8	76.9	97.9	36.62	7.9
PLT < 102 and %Luc > 1.8 and MPV > 14	69.2	98.0	34.60	7.4

All blood smears that were indicated false positive by the combination PLT < 102 and %LUC > 1.8 were re-evaluated microscopically and an additional six *B. canis* and seven *A. phagocytophilum* cases were identified, apparently all subclinical infections. Including these *Babesia* cases, the PV+ would increase to 12.0%. We note that these additional cases were also labeled positive by the machine-learning model described below.

### Machine learning

Models performed very similarly on the cross-validated training data with an AUC of 99.3 (98.7–99.7, 95% CI) for the random forest and almost identical performances for the logistic regression and XGBoost classifiers. Only the decision tree performed slightly worse with an AUC 97.0 (94.8–98.6, 95% CI). Henceforth, results will be presented for the random forest classifier.

**Fig. 2:**
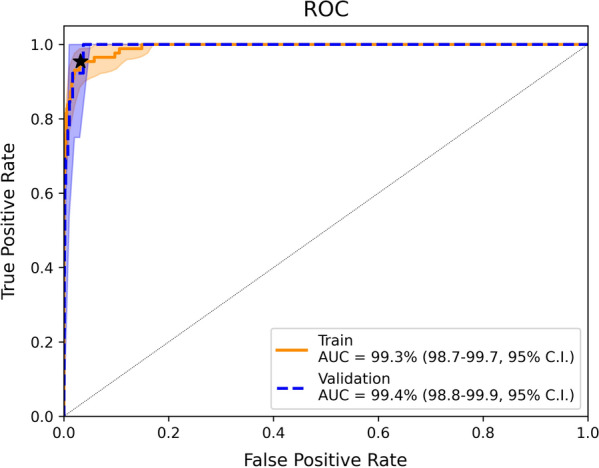
ROC curves from the random forest classifier for the training (orange) and validation (blue) sets. The star represents the model whose performance is referred to in the text (sensitivity of 95% on the training set)

Each classifier was applied to the validation data where similar performance was observed. The random forest classifier had an AUC of 99.4 (98.7–99.8, 95% CI), indicating that the model generalizes well (see Table 6; Fig. 2). A confusion matrix, using the threshold resulting in 95% sensitivity in the training dataset, is shown in Table 7. In the validation dataset we observe a sensitivity of 100%, specificity of 95.7 (95.1–96.2, 95% CI) and positive-predictive value (PV+) of 5.1 (2.5–7.9, 95% CI). Note that the PV+ depends on the prevalence of positive samples in the dataset, which differs between training and validation data.

**Table 6:** Comparison of the model performance on the train and validation set. For the computation of the sensitivity and specificity the threshold for each model for positive predictions was chosen such that the sensitivity on the training set is 95%

Model	Train			Validation		
	AUC (%)	Sensitivity (%)	Specificity (%)	AUC (%)	Sensitivity (%)	Specificity (%)
Conventional statistics (rule-based)	93.7 (90.1–96.6, 95% CI)	89.7 (82.5–95.6, 95% CI)	97.7 (96.9–98.6, 95% CI)	91.1 (80.6–98.9, 95% CI)	84.6	97.7 (97.3–98.1, 95% C.I
Decision tree	97.0 (95.0–98.6, 95% CI)	95.4 (90.7–99.7, 95% CI)	89.1 (87.2–90.8, 95% CI)	98.0 (96.7–99.0, 95% CI)	100	87.0 (86.1–87.8, 95% CI)
Logistic regression	99.3 (98.8–99.7, 95% CI)	95.4 (90.5–98.9, 95% CI)	96.8 (95.6–97.7, 95% CI)	98.8 (98.3–99.2, 95% CI)	100	89.7 (88.8–90.5, 95% CI)
Random forest	99.3 (98.6–99.7, 95% CI)	95.4 (90.3–98.9, 95% CI)	96.9 (95.9–97.8, 95% CI)	99.4 (98.8–99.8, 95% CI)	100	95.7 (95.1–96.2, 95% CI)
XGBoost	99.3 (98.8–99.8, 95% CI)	95.4 (90.6–99.0, 95% CI)	96.8 (95.7–97.7, 95% CI)	99.4 (98.5–99.9, 95% CI)	100	93.7 (93.1–94.3, 95% CI)

**Table 7:** Confusion matrix for the validation data set. Algorithm predictions are from the random forest model

		Predicted label	
		*B. canis*	*B. canis* +
True label	*B. canis* −	5405	244
	*B. canis* +	0	13

**Fig. 3:**
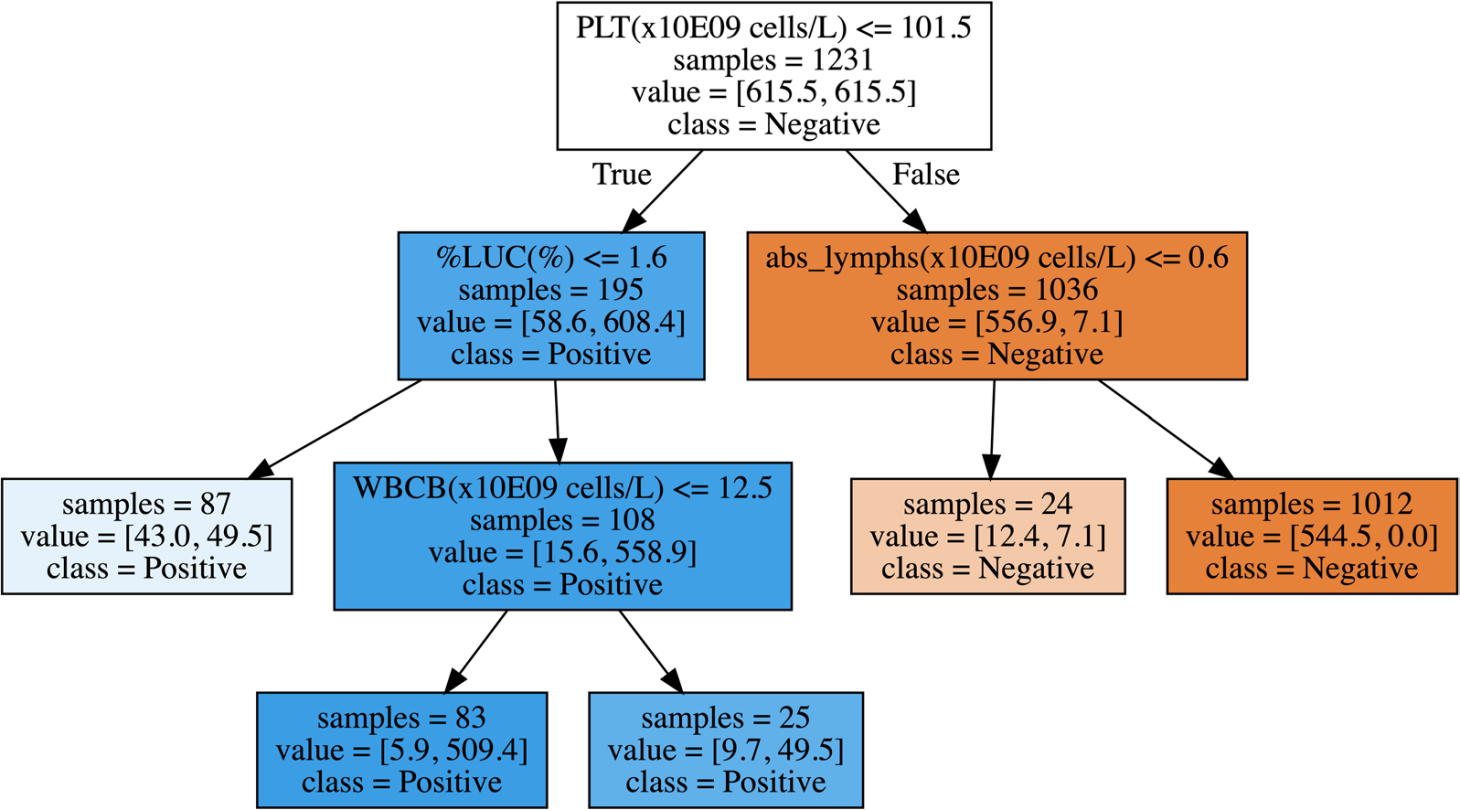
Decision tree classifier. The top line shows the condition for descending the tree. Blue leaves imply the model predicts a positive *B. canis* infection, whereas orange leaves predict no infection. Samples refers to the total number of samples from the training set that end up in a particular leaf. Values are the weighted samples in a leaf, where the first entry corresponds to the negative samples (which have a weight of ~ 0.54) and the second entry to the positive samples (with a weight of ~ 7.07). Whichever value is largest determines the leaf label. Note that the complete right branch only contains one positive sample in the train set. As such, the parameter *abs_lymphs(*× *10E9 cells/l)* is plausibly of lesser importance, despite it being high up in the tree

**Fig. 4:**
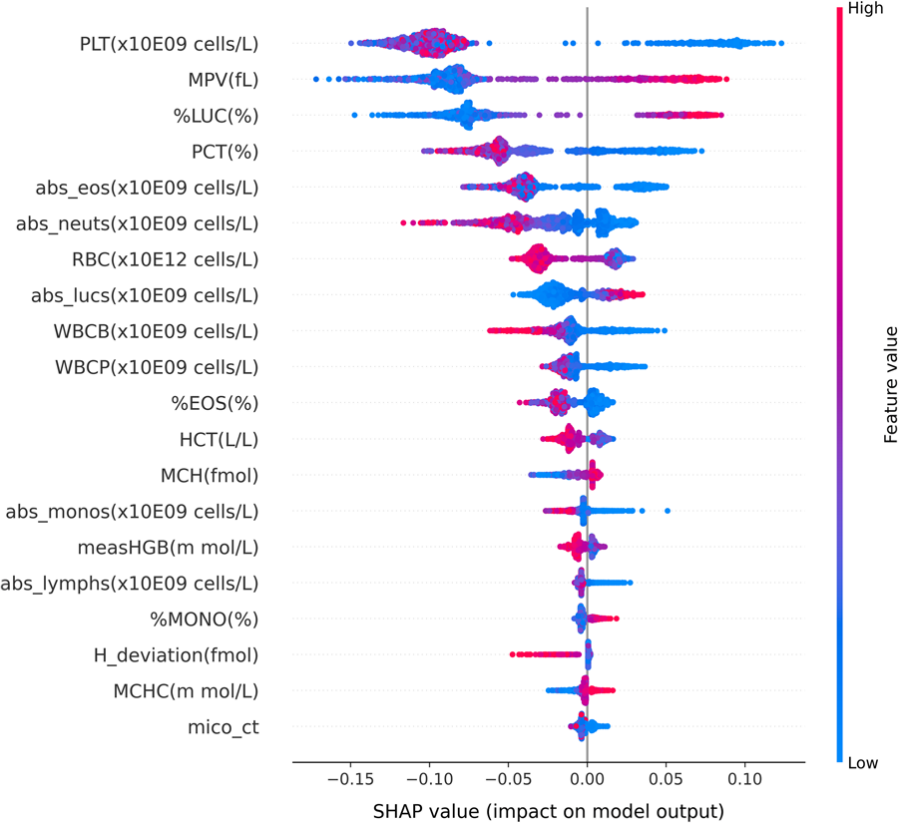
Feature importances of the random forest model determined on the train set in descending order. Each dot is a data point. The SHAP value indicates how much that feature contributes to the prediction of that data point, where large deviations from zero mean a larger contribution and positive values contribute toward a positive prediction of *B. canis*. Red (blue) colored dots refer to high (low) feature values. For instance, high values (red dots) of the feature *%LUC(%)* make it more likely a dog is infected with *B. canis* (positive SHAP value)

The advantage of the decision tree algorithm is its interpretability. Figure 3 shows our best decision tree algorithm. At each node one can see what feature and which value for that feature determines the split. For instance, this tree shows that patients with a small number of platelets are more likely to have *B. canis* parasitemia. Due to the larger number of trees, and the random selection of features when growing the trees, the random forest is less interpretable than the decision tree. However, using an approach like SHapley Additive exPlanations (SHAP) [19], an insight can be gained into what are the most important features driving the prediction. For the random forest we find, in descending order of importance, the following ADVIA parameters to drive the decision PLT(× 10E9 cells/l), MPV(fl), %LUC(%), platelet concentration (PCT) (%), absolute count of eosinophils (abs_eos) (× 10E9 cells/l) and absolute count of neutrophils (abs_neuts) (× 10E9 cells/l) (see Fig. 4).

## Discussion

In this study we identified hematological parameters suggestive of *B. canis* parasitemia in dogs by using conventional statistics and data analysis as well as machine learning. Both methods identified the same important parameters (PLT, MPV, %LUC), while the random forest used additional parameters which were of lesser importance to the model. We were able to formulate a conventional statistics (CS) and machine learning (ML) model to screen for *Babesia* parasitemia in dogs with a sensitivity of 84.6% (CS) and 100% (ML), a specificity of 97.7% (CS) and 95.7% (ML) and a positive likelihood ratio (LR+) of 36.78 (CS) and 23.2 (ML). Because we considered the use of this model primarily as a screening tool, we preferred a high sensitivity. Note that the sensitivity of the random forest model is 100% on the validation set. However, due to the limited number of positive samples (13), it is likely slightly lower, as indicated by the cross-validation results on the training dataset, which shows 90–99% at 95% CL (Table 6).

This is to our knowledge the first veterinary study that describes the use of ML to identify an infectious disease in readily available data (ADVIA hematology parameters) of dogs.

That a decreased platelet count (PLT) was important in our model for identification of parasitemia is in accordance with previous studies reporting thrombocytopenia in *Babesia* infections [21–25]. The underlying mechanism for thrombocytopenia in canine babesiosis is not yet defined. Possible reasons are an immune-mediated destruction of the platelets and an increased consumption [24], co-infections with *Ehrlichia* spp. [26] or formation of platelet aggregates [27]. Thrombocytes appear to also play a major role in the response to human erythrocyte *Plasmodium falciparum* infections (malaria). Platelets can protect against malaria progression by binding to infected erythrocytes and induce killing mechanisms. Also, platelets can promote sequestration of infected erythrocytes [28]. It is not known whether these mechanisms also occur in erythrocyte infections with *B. canis*.

Another important parameter was an increased mean platelet volume (MPV), and this was also found by Furlanello et al. and de Gopegui et al. [23, 24]. A rather surprising finding was the importance of an increase in large unstained cells (%LUC) in our models. The %LUC represents young or transformed lymphocytes. This could relate to the lymphopenia that is found in *B. canis* [21, 24, 29] and can be a sign of regeneration. Studies on hematologic changes in human malaria infections have also shown that the most common change was an increase in %LUC and thrombocytopenia [30], comparable with our findings in *B. canis* parasitemia.

The use of machine learning algorithms as a diagnostic tool in veterinary medicine is increasing in popularity. Recently, papers were published on hypoadrenocorticism [13], hyperadrenocorticism [15], early chronic kidney disease [31] and general chronic kidney disease [32]. As demonstrated in this work, it can be used as a tool to automatically uncover patterns in datasets. The decision tree model shows many similarities to the conventional statistics model in its decision logic and performance, but it determined the *if-then* rules automatically. In addition, more complex models such as the random forest and XGBoost model can capture more complicated (non-linear) relationships, which is reflected in the improved performance over the simpler decision tree model.

Important limitations of our study are that *Babesia* parasitemia was studied, not *Babesia* infections in general. PCR analysis would have been the most reliable method to diagnose a *B. canis* infection. As PCR analysis was not performed on all of the nearly 7000 samples, false-negative ground-truth labels were possible in our dataset. This potentially affects our models’ performances. Re-evaluation of a number of samples showed that it is possible that some of the false-positive predictions turn out to be actual positives that were initially missed, resulting in an underestimate of the PV+. In addition, PCR analysis still probably outperforms our models in terms of sensitivity for detecting canine babesiosis.

Another important limitation in our study is that an artificial prevalence was used because of the low natural prevalence of *B. canis* infections in The Netherlands. This was done by collecting positive samples from a longer period of time and introducing them to the dataset of all complete blood counts that were performed in our laboratory during a 3-month period. We used this artificial prevalence to train our models on a dataset that would be more comparable to the situation in endemic areas.

## Conclusions

This study introduces two methods of screening for *B. canis* parasitemia on readily available complete blood count data from ADVIA hematology systems. The algorithms can be easily introduced in laboratories that use these popular hematology systems. According to our current findings with a likelihood ratio of 37, when the algorithm marks a sample as ‘suggestive’ for *Babesia* parasitemia, the sample is > 37 times more likely to show *Babesia* merozoites on blood smear analysis.

## Data Availability

The datasets used and/or analyzed during the current study are available from the corresponding author on reasonable request. Analysis code is publicly available via GitHub: https://github.com/richardbartels/babesia-canis-hematology.

## Abbreviations

|RBC (× 10E12 cells/L): Red blood cell count
HGB (m mol/L): Hemoglobin concentration
|HCT (L/L)|: Hematocrit
|%LUC (%)|: % Large unstained cells
MN_y_peak ([No Units]): Peak Y channel of Mononuclear cluster
lob_Index ([No Units]): Lobularity index
pcnt_low_retics (%): % Of low absorbance reticulocytes with absorption values falling between RTC threshold and low/medium RTC
pcnt_med_retics (%): % Of medium absorbance reticulocytes with absorption values falling between low/medium RTC threshold and medium/high RTC threshold
retics_cells_thresh ([No Units]): Reticulocyte (RTC) threshold
med_retic_thresh ([No Units]): Threshold for medium intensity staining reticulocytes in absorption cytogram
high_retic_thresh ([No Units]: Threshold for high intensity staining reticulocytes in absorption cytogram
retic_MCV (fL): MCV of reticulocytes
retic_HDW (m mol/L): HDW of reticulocytes
retic_H_mean (fmol: Mean Hb content of reticulocytes
% abnormal_cells ([No Units]): % Of total cells that exceed the abnormal limit number of standards deviations from the center of the cluster to which they were assigned
pcnt_high_px (%): % Cells with high peroxidase absorption values
Lymph_noise_valley: Lymph noise valley
|IRF-M + H (%)|: Immature reticulocyte fraction for medium + high absorbance reticulocytes
|MCV_rm_delta (fL)|: Calculated difference between the MCV values of the reticulocytes and mature cell populations
HDW_rm_delta (m mol/L): Calculated difference between the HDW values of the reticulocytes and mature cell populations
|CH_rm_delta (fmol)|: Calculated difference between the Hb content values of the reticulocytes and mature cell populations
CHDW_rm_delta (fmol): Calculated difference between the Hb content distribution width values of the reticulocytes and mature cell populations
|%macro_r ([No Units])|: % Macrocytic reticulocytes
%lowCH_m ([No Units]): % Mature cells with low Hb content
|%highCH_r ([No Units])|: % Reticulocytes with high Hb content
|RBC_2-D_count (× 10E12 cells/L)|: RBC count taken from the 2D-PLT method
PLT (× 10E09 cells/L): Platelet count
MPV (fL: Mean platelet volume
|MPC (g/L)|: Mean platelet component content
PCDW (g/L): Platelet component concentration distribution width
MPM (pg): Mean platelet dry mass
|PMDW (pg)|: Platelet dry mass distribution width
RBC_Ghosts (× 10E12 cells/L): RBC ghost cell count
BaroxNRBCCount ([No Units]): Number of nucleated red blood cells according to the Barox count
endCurveMu ([No Units]): Mu of gauss fit to LUCs section of NRBC histogram
